# MIND diet lowers risk of open-angle glaucoma: the Rotterdam Study

**DOI:** 10.1007/s00394-022-03003-w

**Published:** 2022-09-20

**Authors:** Joëlle E. Vergroesen, Tosca O. E. de Crom, Cornelia M. van Duijn, Trudy Voortman, Caroline C. W. Klaver, Wishal D. Ramdas

**Affiliations:** 1 Department of Ophthalmology, Erasmus MC University Medical Center, PO Box 2040, 3000 CA Rotterdam, The Netherlands; 2 Department of Epidemiology, Erasmus MC University Medical Center, PO Box 2040, 3000 CA Rotterdam, The Netherlands; 3 Nuffield Department of Population Health, University of Oxford, Oxford, OX3 7LF UK; 4 Division of Human Nutrition and Health, Wageningen University & Research, PO Box 17, 6700 AA Wageningen, The Netherlands; 5 Department of Ophthalmology, Radboud University Medical Center, PO Box 9101, 6500 HB Nijmegen, The Netherlands; 6 Institute of Molecular and Clinical Ophthalmology, University of Basel, Basel, Switzerland

**Keywords:** Open-angle glaucoma, Intraocular pressure, Neurodegeneration, MIND diet, Mediterranean diet, Dutch dietary guidelines

## Abstract

**Purpose:**

To assess the association between the Mediterranean-DASH Intervention for Neurodegenerative Delay (MIND) diet and the incidence of open-angle glaucoma (iOAG), as well as the association between iOAG and two other well-established diets in the Netherlands, i.e., the Mediterranean diet and Dutch dietary guidelines.

**Methods:**

In the Rotterdam Study, participants were followed for iOAG since 1991, with intervals of approximately 5 years. A total of 170 participants developed iOAG during follow-up. Participants with iOAG were matched with healthy controls on age and sex in a case:control ratio of 1:5. The associations between food frequency questionnaire-derived diet adherences (baseline) and iOAG were analyzed using multivariable conditional logistic regression analyses. The associations between the diet adherences and intraocular pressure (IOP; a risk factor for OAG) were assessed using multivariable linear regression analyses.

**Results:**

Greater adherence to the MIND diet was associated with a decreased iOAG risk (odds ratio [95% confidence interval]: 0.80 [0.66 to 0.96], for each 10-percent increase in adherence). Food component analyses showed that, in particular a higher intake of green leafy vegetables, berries and fish tended to be protective for iOAG. No significant associations were observed between adherence to the Mediterranean diet or Dutch dietary guidelines and iOAG. Moreover, none of the three examined diets were associated with IOP.

**Conclusion:**

Adherence to the MIND diet was significantly associated with a lower incidence of OAG in contrast to adherence to the Mediterranean diet or the Dutch dietary guidelines. As this association was IOP-independent, the MIND diet may be particularly relevant for the prevention of neurodegeneration in the eye.

**Supplementary Information:**

The online version contains supplementary material available at 10.1007/s00394-022-03003-w.

## Introduction

Glaucoma is a debilitating neurodegenerative eye disease that causes irreversible blindness. It currently affects more than 80 million people worldwide, among whom approximately 11 million are bilaterally blind [1]. The disease is characterized by visual field loss typically starting in the periphery. Subsequently, the center visual field gets affected, resulting in a low visual acuity, which cannot be restored. Early symptoms are often not noticed by the patient, while early detection and intervention is crucial to slow down disease progression. A high intraocular pressure (IOP) is an important modifiable risk factor to target the progressive loss of retinal ganglion cells in glaucoma. However, it is likely that IOP-independent mechanisms play a role as well: between one-third and one-half of eyes with glaucoma have no elevated IOP [2], suggesting that neurodegeneration is the primary cause of the disease. Further, visual field deterioration is found to continue in 30 to 40% of the patients despite a reduction of the IOP [3, 4].

A higher intake of certain dietary components, including tea [5], fruits, and vegetables [6, 7], among which green leafy vegetables specifically [8–10], have been linked to lower IOP levels and lower incidence of glaucoma. This is mainly because they contain high concentrations of antioxidants and flavonoids, and thereby have anti-inflammatory and neuroprotective properties. However, individuals consume a whole diet instead of isolated dietary components and interactions among components may origin potential protective effects. Most research in the field of nutrition, therefore, focuses on dietary patterns, e.g., the Mediterranean diet or national dietary guidelines, but for glaucoma such research is limited.

Recently, the Mediterranean-DASH Intervention for Neurodegenerative Delay (MIND) diet was developed as a strategy to promote healthy cognitive ageing [11]. It is a combination of the Mediterranean diet [12] and the Dietary Approaches to Stop Hypertension (DASH) diet [13] and has been associated with reduced incidence of Alzheimer’s disease [14, 15] and slowed cognitive decline [11, 16]. The eye and brain have a shared embryonic origin, as the retina and optic nerve extend from the diencephalon during embryonic development [17]. As such, despite their diverse morphology, retinal ganglion cells display the typical properties of central nervous system neurons. Additionally, open-angle glaucoma (OAG) and Alzheimer’s disease share multiple common biochemical and pathological changes [18]. It is, therefore, likely that the MIND diet has neuroprotective effects in not only the brain but also the eye.

We determined the association between adherence to the MIND diet, the Mediterranean diet [12], and the Dutch dietary guidelines [19, 20], and incidence of OAG (iOAG). To assess whether potential associations with iOAG are IOP-dependent, we also examined the associations between adherence to these three dietary patterns and IOP.

## Materials and methods

### Ethics statement

The Rotterdam Study has been approved by the Medical Ethics Committee of Erasmus MC (registration number MEC 02.1015) and by the Dutch Ministry of Health, Welfare, and Sport (Population Screening Act WBO, license number 1071272-159521-PG). The Rotterdam Study has been entered into the Netherlands National Trial Register (NTR; www.trialregister.nl) and into the WHO International Clinical Trials Registry Platform (ICTRP; www.who.int/ictrp/network/primary/en/) under shared catalog number NTR6831. All participants provided written informed consent following the declaration of Helsinki to participate in the study and to have their information obtained from their treating physicians.

### Study population

Participants were derived from three independent cohorts from the prospective population-based Rotterdam Study (RS-I, RS-II, RS-III), designed to assess determinants of age-related diseases in the middle-aged and elderly population (45+ years). Enrollment for the ophthalmic part started in 1991; after the baseline visit, participants were invited for follow-up visits with intervals of approximately 5 years [21]. Of the 8679 participants with ophthalmic and iOAG examinations, 6941 had baseline data on dietary intake. Of those, 170 participants developed iOAG during follow-up. Since age has a strong positive association with iOAG risk [22] and strong inverse association with dietary intake [23, 24], and dietary intake is different for females compared to males [25], we chose a nested matched case–control design for the analyses. We matched cases and controls on age (with a maximum difference of 3 years) and sex in a 1:5 ratio, and sampled without replacement. The final dataset consisted of 170 cases and 850 controls.

### Ophthalmic assessment

The eye examinations included Goldmann applanation tonometry (Haag-Streit AG, Bern, Switzerland) and visual field testing (Humphrey Field Analyzer; HFA II 740; Carl Zeiss, Oberkochen, Germany). iOAG was defined as glaucomatous visual field loss in at least one eye with reproducibility of the defect, independent of IOP [26]. Other possible causes of visual field loss were excluded. iOAG cases had an open anterior chamber angle and no history or signs of secondary glaucoma [26]. For IOP, three measurements were taken from each eye, of which the median value was recorded. For iOAG cases, we used IOP measurements of the affected eye. If both eyes were affected or unaffected, a random eye was selected.

### Dietary data and diet scoring

Dietary intake was assessed at baseline using food frequency questionnaires (FFQs) as described in detail elsewhere [27]. Briefly, for the first two cohorts, a 170-item FFQ was applied as two-stage approach. First, participants indicated which foods they consumed at least twice a month in the preceding year. Second, a trained dietician used this list to identify how often and in which amounts the foods were consumed. For the third cohort, dietary intake data was collected using an extended self-administered 389-item FFQ. Both FFQs were previously validated and showed reasonable to good estimates of nutrient intake [28–30]. All food items were assessed based on the frequency of consumption (in times per month or per week), the number of servings per day (expressed in standardized household measures) as well as on the preparation methods. Participants with unreliable reported dietary intake (energy intake < 500 kcal/day or > 5000 kcal/day) were excluded. The MIND diet contains recommendations regarding 15 food components, including 10 food components considered as brain-healthy (i.e., green leafy vegetables, other vegetables, nuts, berries, beans, whole grains, fish, poultry, olive oil, and wine) and five food components considered as unhealthy (i.e., red meat, butter and stick margarine, cheese, pastries and sweets, and fried/fast food) [11]. Scoring was performed based on non-adherence (0), moderate adherence (0.5) and good adherence (1). In the case of olive oil, it was considered good adherence when olive oil was used as the primary cooking fat (> 50%) and non-adherence when used less than or equal to 50%. The final MIND diet score ranged from 0 to 15. The Mediterranean diet contains recommendations regarding 11 food components: vegetables, fruits, legumes, whole grains, fish, full-fat dairy products, potatoes, olive oil, poultry, meat, and alcoholic beverages [12]. Adherence to each food component was scored from 0 to 5, with 5 being the greatest adherence. The final Mediterranean diet score ranged from 0 to 55. The Dutch dietary guidelines (2015) include recommendations for 14 food components: vegetables, fruits, whole grains, legumes, nuts, fish, tea, dairy products, whole grains of total grains, unsaturated fats and oils of total fats, red and processed meat, sugar-containing beverages, alcohol, and salt [19, 20]. Participants were scored as non-adherent (0) or adherent (1), resulting in a final Dutch dietary guidelines score ranging from 0 to 14. Diet adherence was expressed in percentage, calculated by dividing the adherence score by the maximum adherence score theoretically possible and multiplying by 100. E.g., a MIND diet score of 8.5 would translate into 56.7% adherence to the MIND diet.

### Covariates

Education level was assessed with questionnaires and categorized into: primary education (with or without a partially completed higher education), lower education (lower vocational or lower secondary education), intermediate education (intermediate vocational or general secondary education), or higher education (higher vocational education or university). Smoking status was obtained using questionnaires and participants were classified as non-smoker, former smoker, or current smoker. Weight and height were measured at the research center. Body mass index (BMI) was calculated as weight in kilograms divided by height in meters squared. Energy intake was obtained from the previously described FFQs. For physical activity two different questionnaires were used: a validated adapted version of the Zutphen Physical Activity Questionnaire [31] (RS-I and RS-II) and the LASA Physical Activity Questionnaire (RS-III) [32]. Data were recalculated into metabolic equivalent of task (MET)-hours per week. The total activity scores from these questionnaires are not one on one comparable, and we, therefore, used a cohort specific *z*-standardized score.

### Statistical analyses

Differences in baseline characteristics were evaluated using chi-squared tests and independent-samples *t* tests. One-way ANOVA was used to compare the baseline characteristics of participants in the different quartiles of the diet adherences. To determine the association between adherence to the different diets and iOAG, we performed multivariable conditional logistic regression analyses to calculate odds ratios (ORs) with corresponding 95% confidence intervals (CIs). Additionally, we modelled the diet adherences in quartiles with the first quartile (Q1) as reference category to test for evidence of linear trends. The median value for each category as continuous variables was used in separate conditional logistic regression models. The final models included BMI, energy intake, physical activity, and follow-up time (all as continuous variables). Follow-up duration was calculated from baseline until the last visit with reliable ophthalmic examination or the first visit with iOAG diagnosis. To assess potential reverse causality, we analyzed the association between the MIND diet and iOAG in cumulative follow-up intervals. Moreover, to evaluate whether one individual component of the MIND diet explained the potential association, we repeated the analysis with versions of the MIND diet where adherence to an individual food component was one at the time excluded from the total adherence. If associations substantially changed after excluding a single component, the association of this individual component (in grams per day, week, or month, whichever was applicable) with iOAG was determined in post hoc analyses using multivariable conditional logistic regression analyses. Additionally, we observed the effect of including education level and smoking status (lifestyle factors affecting nutrition quality) or IOP (potential mediator in the association with iOAG) in the models. The dose–response relation between adherence to the MIND diet and predicted iOAG probability was examined using generalized additive modelling. Moreover, the association between adherence to the different diets and  IOP at follow-up was assessed by performing multivariable linear regression analyses, adjusting for the same covariates as mentioned above. Statistical analyses were performed using SPSS v25.0 (SPSS Inc., Chicago, IL, USA) and R v3.6.1 (www.r-project.org), with packages ggplot2, foreign, mgcv, tibble, dplyr, ggpubr and DescTools. A *p* value < 0.05 was considered statistically significant.

## Results

The baseline characteristics of cases and controls are displayed in Table 1. Participants with iOAG had a lower BMI and higher IOP. Adherence to the MIND diet was significantly lower in cases than controls, but adherence to the Mediterranean diet and Dutch dietary guidelines were not. Baseline characteristics according to quartiles of MIND diet adherence are presented in Table 2. Participants with a greater MIND diet adherence were younger, more often female and had a higher education level (Table 2). Similar patterns were observed for participants with a greater Mediterranean diet adherence (Supplementary Table 1) and greater Dutch dietary guidelines adherence (Supplementary Table 2).

**Table 1:** Baseline characteristics of participants that did and did not develop incident open-angle glaucoma during follow-up

	No iOAG (*N* = 850)	iOAG (*N* = 170)	*P* value
Age (years)	64.9 (7.0)	65.8 (6.9)	0.15
Female sex, *N* (%)	460 (54.1)	92 (54.1)	1.00
Education, *N* (%)			0.39
Primary education	106 (12.5)	20 (11.8)	
Lower education	364 (42.8)	78 (45.9)	
Intermediate education	231 (27.2)	52 (30.6)	
Higher education	141 (16.6)	20 (11.8)	
Smoking status, *N* (%)			0.96
Non-smoker	260 (30.6)	54 (31.8)	
Former smoker	404 (47.5)	80 (47.1)	
Current smoker	183 (21.5)	36 (21.2)	
BMI (kg/m^2^)	27.0 (4.1)	25.9 (3.3)	< 0.001*
Energy intake (kcal/day)	2104.7 (577.1)	2054.3 (515.0)	0.29
Physical activity (MET h/week)	0.0 (0.9)	0.1 (0.9)	0.18
IOP (mmHg)	14.2 (3.0)	16.4 (3.9)	< 0.001*
Follow-up time (years)	9.7 (4.9)	10.9 (5.3)	0.003*
MIND diet adherence (%)^a^	43.5 (10.7)	41.5 (8.5)	0.008*
Mediterranean diet adherence (%)^b^	59.3 (8.0)	58.5 (7.1)	0.23
Dutch dietary guidelines adherence (%)^c^	47.7 (13.5)	49.7 (13.9)	0.04*

Data are presented as mean (standard deviation), unless stated otherwise

*iOAG* incident open-angle glaucoma, *N* number, *SD* standard deviation, *BMI* body mass index, *MET* metabolic equivalent of task, *IOP* intraocular pressure, *MIND* Mediterranean-DASH Intervention for Neurodegenerative Delay

*P < 0.05

^a^Percentage calculated from theoretical score range: 0–15

^b^Percentage calculated from theoretical score range: 0–55

^c^Percentage calculated from theoretical score range: 0–14

**Table 2:** Baseline characteristics of participants by adherence to the MIND diet (per quartiles)

	Q1 (*N* = 338)	Q2 (*N* = 146)	Q3 (*N* = 343)	Q4 (*N* = 193)	*P* ANOVA
iOAG, *N* (%)	62 (18.3)	26 (17.8)	61 (17.8)	21 (10.9)	0.12
Age (years)	66.6 (7.4)	65.8 (6.6)	64.6 (7.0)	62.5 (5.6)	< 0.001*
Female sex, *N* (%)	157 (46.4)	66 (45.2)	199 (58.0)	130 (67.4)	< 0.001*
Education, *N* (%)					< 0.001*
Primary education	46 (13.6)	19 (13.0)	43 (12.5)	18 (9.3)	
Lower education	149 (44.1)	66 (45.2)	156 (45.5)	71 (36.8)	
Intermediate education	94 (27.8)	40 (27.4)	98 (28.6)	51 (26.4)	
Higher education	44 (13.0)	19 (13.0)	45 (13.1)	53 (27.5)	
Smoking status, *N* (%)					0.05*
Non-smoker	101 (29.9)	41 (28.1)	107 (31.2)	65 (33.7)	
Former smoker	148 (43.8)	65 (44.5)	170 (49.6)	101 (52.3)	
Current smoker	87 (25.7)	39 (26.7)	66 (19.2)	27 (14.0)	
BMI (kg/m^2^)	26.6 (4.0)	26.4 (3.2)	27.1 (4.1)	27.0 (4.3)	0.21
Energy intake (kcal/day)	2139.6 (551.5)	2161.1 (551.6)	1994.3 (549.3)	2152.4 (615.7)	< 0.001**
Physical activity (MET h/week)	0.0 (1.0)	0.1 (0.9)	0.1 (0.9)	0.1 (0.9)	0.14
IOP (mmHg)	14.6 (3.3)	14.2 (3.1)	15.0 (3.2)	14.1 (3.3)	0.005*
Follow-up time (years)	10.8 (5.0)	9.9 (4.8)	10.1 (5.0)	8.0 (4.5)	< 0.001*
MIND diet adherence (%)^a, b^	32.2 (4.6)	40.0 (0.0)	46.5 (2.8)	58.8 (6.1)	< 0.001*

Data are presented as mean (standard deviation), unless stated otherwise

*Q* quartile, *ANOVA* analysis of variance, *iOAG* incident open-angle glaucoma, *N* number, *SD* standard deviation, *BMI* body mass index, *MET* metabolic equivalent of task, *IOP* intraocular pressure, *MIND* Mediterranean-DASH Intervention for Neurodegenerative Delay

*P < 0.05

^a^Percentage calculated from theoretical score range: 0–15

^b^Range: Q1: 16.7–36.7%, Q2: 36.7–43.3%, Q3: 43.3–53.3%, Q4: 53.3–80.0%

In the multivariable-adjusted model (Table 3, model 1), each 10-percent increase in MIND diet adherence was associated with a 20% reduction in the risk of iOAG (OR [95% CI]: 0.80 [0.66 to 0.96]). When analyzing the cumulative follow-up intervals, a greater adherence to the MIND diet was associated with lower iOAG risk during every cumulative follow-up interval after 10 years follow-up (Supplementary Fig. 1). There were no significant associations between adherence to the Mediterranean diet or the Dutch dietary guidelines and iOAG (Table 3).

**Table 3:** Multivariable-adjusted odds ratios with corresponding 95% confidence intervals for incident open-angle glaucoma per 10-percent increase in diet adherence

	Model 1		Model 2		Model 3	
	Odds ratio	*P* value	Odds ratio	*P* value	Odds ratio	*P* value
MIND diet adherence	0.80 (0.66; 0.96)	0.02*	0.80 (0.67; 0.97)	0.02*	0.79 (0.65; 0.97)	0.02*
Mediterranean diet adherence	0.90 (0.72; 1.14)	0.39	0.90 (0.71; 1.14)	0.37	0.94 (0.73; 1.20)	0.60
Dutch dietary guidelines adherence	1.08 (0.95; 1.22)	0.23	1.08 (0.95; 1.23)	0.24	1.08 (0.95; 1.24)	0.23

Model 1: adjusted for body mass index, energy intake, physical activity, and follow-up time. Model 2: model 1 additionally adjusted for education level and smoking status. Model 3: model 1 additionally adjusted for intraocular pressure

*MIND* Mediterranean-DASH Intervention for Neurodegenerative Delay

*P < 0.05

**Fig. 1:**
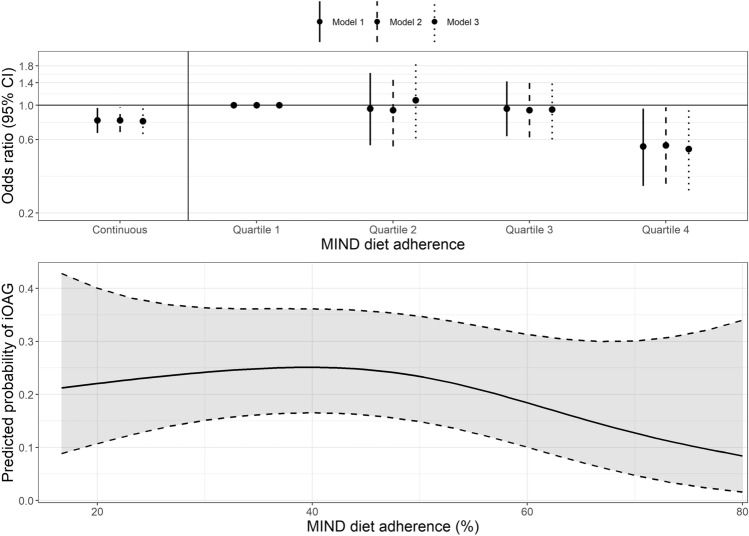
**a** Multivariable-adjusted odds ratios with corresponding 95% confidence intervals (CIs) for incident open-angle glaucoma (iOAG) per 10-percent increase in MIND diet adherence and per quartile. Model 1: adjusted for body mass index, energy intake, physical activity, and follow-up time. Model 2: model 1 additionally adjusted for education level and smoking status. Model 3: model 1 additionally adjusted for intraocular pressure. **b** Graphic presentation of the multivariable-adjusted dose–response relation between adherence to the MIND diet and iOAG. Dotted lines represent 95% CIs. The reference value is the value associated with the mean MIND diet adherence for all participants. *MIND* Mediterranean-DASH Intervention for Neurodegenerative Delay

After adjustment for covariates (Fig. 1a, model 1), participants in the highest quartile of adherence to the MIND diet (Q4: mean adherence 58.8%) had the largest risk reduction (OR [95% CI]: 0.54 [0.30 to 0.95]) compared to participants in the lowest quartile (Q1: mean adherence 32.2%) (*p* trend = 0.07). Figure 1b presents a graphic representation of the dose–response relation between adherence to the MIND diet and iOAG, analyzed in a generalized additive multivariable-adjusted model. There was a non-linear relationship, in which the influence of adherence to the MIND diet improved particularly for adherence greater than 60%, as was also identified in the quartile analysis.

**Fig. 2:**
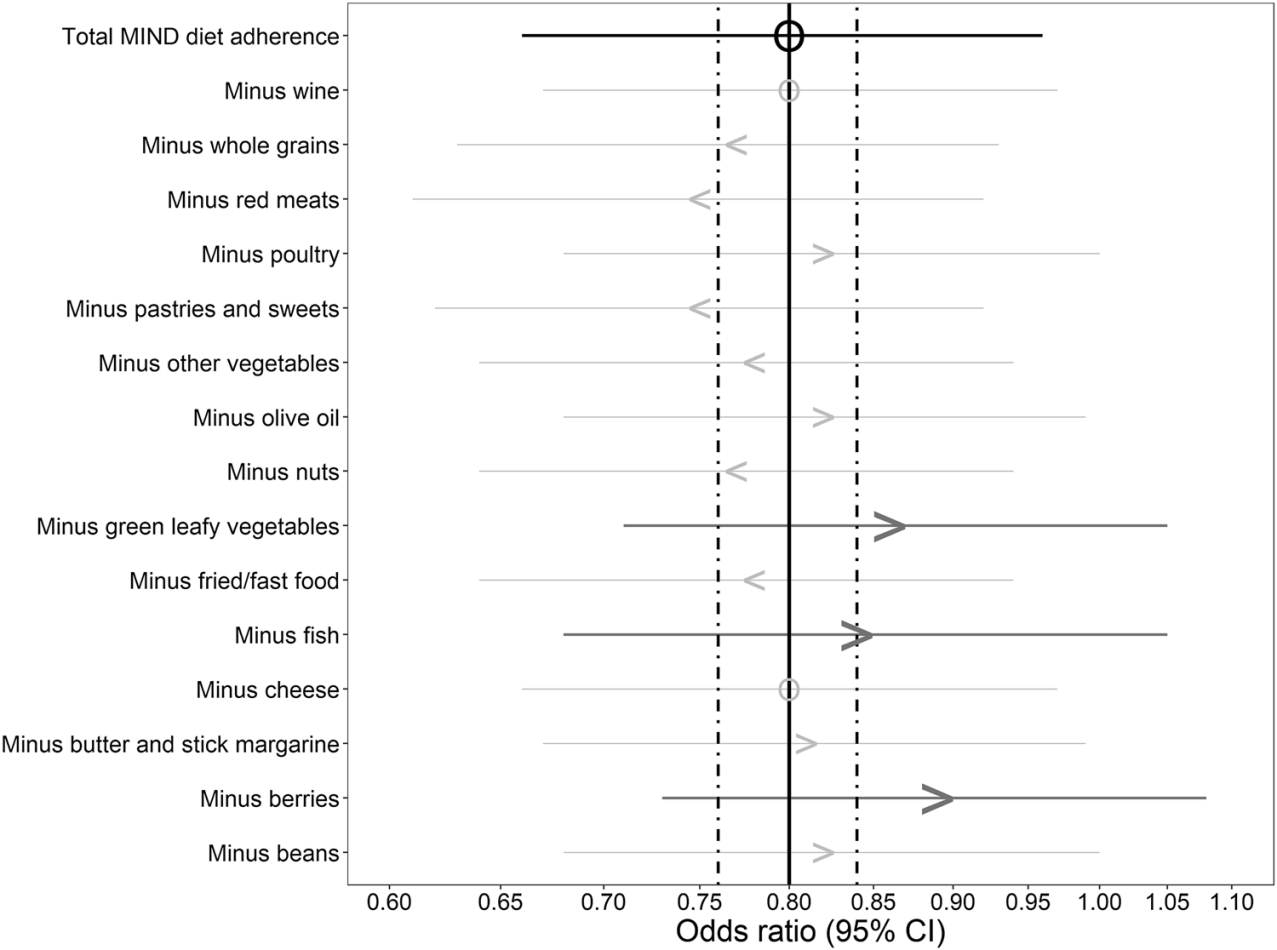
Multivariable-adjusted odds ratios with corresponding 95% confidence intervals (CIs) for incident open-angle glaucoma per 10-percent increase in MIND diet adherence, and food component analyses. Results of model 1 are depicted. Model 1: adjusted for body mass index, energy intake, physical activity, follow-up time and adherence to components of interest. Arrowheads indicate the direction in which the association changed by removing the specific food component as compared to the association with the total MIND diet adherence. Size and color vary according to the corresponding effect size. The two dotted vertical lines represent the cut-off value that was used to assess the effect of excluding a single component from the total MIND diet adherence. *MIND* Mediterranean-DASH Intervention for Neurodegenerative Delay

Food component analyses, in which adherence to components of the MIND diet was excluded one at a time from the total adherence, showed that the association between the MIND diet and iOAG was not driven by one single component. Nevertheless, excluding particularly adherence to green leafy vegetables, berries or fish from the total adherence tended to affect the association (Fig. 2, Supplementary Table 3). When studying green leafy vegetables (mean [interquartile range (IQR)]: 290.5 [150.0 to 364.1] g/week), berries (mean [IQR]: 81.9 [0.0 to 101.0] g/week), and fish (mean [IQR]: 627.0 [35.0 to 879.6] g/month) in association with iOAG (Table 4, model 1), a trend towards a protective effect was observed for green leafy vegetables (OR [95% CI]: 0.82 [0.66 to 1.03] for each 250 g/week higher intake), berries (OR [95% CI]: 0.83 [0.73 to 0.94] for each 50 g/week higher intake), and fish (OR [95% CI]: 0.92 [0.85 to 0.99] for each 250 g/month higher intake).

**Table 4:** Multivariable-adjusted odds ratios with corresponding 95% confidence intervals for incident open-angle glaucoma per 1-unit increase in food component

	Model 1		Model 2		Model 3	
	Odds ratio	*P* value	Odds ratio	*P* value	Odds ratio	*P* value
Green leafy vegetables (250 g/week)	0.82 (0.66; 1.03)	0.08	0.82 (0.66; 1.03)	0.09	0.80 (0.60; 1.01)	0.06
Berries (50 g/week)	0.83 (0.73; 0.94)	0.003*	0.83 (0.73; 0.94)	0.003*	0.86 (0.76; 0.97)	0.02*
Fish (250 g/month)	0.92 (0.85; 0.99)	0.03*	0.92 (0.85; 1.00)	0.04*	0.93 (0.86; 1.01)	0.08

Model 1: adjusted for body mass index, energy intake, physical activity, follow-up time and MIND diet adherence minus the MIND diet adherence of components of interest. Model 2: model 1 additionally adjusted for education level and smoking status. Model 3: model 1 additionally adjusted for intraocular pressure

*MIND* Mediterranean-DASH Intervention for Neurodegenerative Delay

*P < 0.05

In the multivariable-adjusted models, IOP was not significantly associated with adherence to the MIND diet, the Mediterranean diet, or the Dutch dietary guidelines (Table 5). The associations between the main iOAG associated food components (green leafy vegetables, berries and fish) and IOP are displayed in Supplementary Table 4. Interestingly, berries were associated with a significant lower IOP (beta [95% CI]: − 0.11 [− 0.21 to − 0.01] for each 50 g/week higher intake), but no significant associations were found for green leafy vegetables or fish.

**Table 5:** Multivariable-adjusted beta’s with corresponding 95% confidence intervals for intraocular pressure per 10-percent increase in diet adherence

	Model 1		Model 2	
	Beta	*P* value	Beta	*P* value
MIND diet adherence	0.02 (− 0.22; 0.27)	0.85	0.04 (− 0.21; 0.30)	0.75
Mediterranean diet adherence	− 0.25 (− 0.58; 0.07)	0.13	− 0.24 (− 0.57; 0.10)	0.17
Dutch dietary guidelines adherence	− 0.03 (− 0.21; 0.16)	0.78	− 0.01 (− 0.20; 0.18)	0.91

Model 1: adjusted for body mass index, energy intake, physical activity, and follow-up time. Model 2: model 1 additionally adjusted for education level and smoking status

*MIND* Mediterranean-DASH Intervention for Neurodegenerative Delay

All aforementioned analyses were additionally adjusted for education level and smoking status (model 2); however, this did not affect any of the results. The analyses with iOAG as dependent variable were also additionally adjusted for IOP (model 3).

## Discussion

In this nested matched case–control study, greater adherence to the MIND diet was associated with decreased incidence of OAG. The MIND diet was not associated with IOP, suggesting other pathways are involved. Neither adherence to the Mediterranean diet nor adherence to the Dutch dietary guidelines was significantly associated with OAG or IOP. These findings suggest that particularly the MIND diet may serve as a beneficial dietary pattern for healthy eyes.

To our knowledge, we are the first to report an association between the MIND diet and iOAG. However, previous studies investigated the association between its specific components and iOAG. We highlight the components that showed to be protective for iOAG in the present study. Greater intake of green leafy vegetables was associated with a 20 to 30% risk reduction of OAG [8]. Also, compared to consuming ≤ 1 serving of green collards and kale per month, consuming > 1 serving per week (OR [95% CI]: 0.43 [0.21 to 0.85]) or ≥ 1 serving per month (OR [95% CI]: 0.31 [0.11 to 0.91]) reduced glaucoma risk [9, 10]. Also in our analyses, an inverse association was observed between green leafy vegetables and iOAG, most likely caused by the presence of high concentrations of nitrate [33], vitamin A, B2, B9, C and E [34], lutein, and zeaxanthin [35].

Higher fruit intake has previously been associated with lower OAG risk [9, 36, 37], but the effect of berries specifically has never been determined. Berries are a good dietary source of bioactive compounds with antioxidant properties, such as vitamins and minerals [38, 39]. They are rich in manganese, vitamin C, and vitamin B9 [40]. Fibers, known to decrease incidence of several types of diseases, are also highly present in berries [41]. Moreover, berries are a remarkably good source of polyunsaturated fats, which are necessary for building cell membranes and covering nerves as well as for proper blood clotting, muscle movement, and protection against inflammation [42]. Additionally, berries have a substantial amount of omega-6 linoleic acid, omega-9 oleic acid, and omega-3 linolenic acid, which are linked to an array of health benefits [43, 44]. Lastly, they are rich in polyphenols [45], which harbor anti-proliferative, anti-diabetic, anticancer, anti-microbial, anti-inflammatory, and antiviral abilities, along with high antioxidant capacity [45–53]. The protective effect of berries in the present study may be explained by the fact that oxidative stress, inflammation, and ocular hypertension play a role in the pathophysiology of OAG [54–56]. The present study confirmed the IOP-lowering potential of berries.

Fatty fish is rich in omega-3 and omega-6 fatty acids, known for their anti-inflammatory, anti-angiogenic, antithrombotic, hypolipidemic, and vasodilatory functions [57]. Inconclusive findings have been reported for the association between fish consumption and OAG. Renard et al. performed a matched case–control study with 334 OAG cases and reported a higher OR with lower fatty fish consumption (OR [95% CI]: 2.14 [1.10 to 4.17]) [58]. Kinouchi et al. observed no significant association between fish consumption and OAG [59], but their number of cases (*N* = 42) was limited. Although the significant association between fish intake and iOAG observed in the present study confirms some of the earlier findings, the effect of fish intake on iOAG remains unclear.

No significant association between adherence to the Mediterranean diet or the Dutch dietary guidelines and OAG or IOP was observed. One previous study reported moderate adherence to the Mediterranean diet in OAG patients; however, they could not address causality since exposure was not measured before onset of disease [60]. Another prospective cohort study reported a lower risk of glaucoma in participants adhering to a Mediterranean lifestyle, but no significant association was found with the Mediterranean diet in the single component analysis [61]. The Dutch dietary guidelines have previously not been assessed with regard to iOAG risk. Although the MIND diet and the other diets share a similar profile, the MIND diet has a separate category for green leafy vegetables and berries. Both have been suggested to have neuroprotective effects [62]. Separating green leafy vegetables from the other vegetables and assessing berries specifically instead of fruit as a whole, may explain why we did find a significant association between the MIND diet and iOAG, but not for the other two diets.

Given that individual caloric intake is relatively stable over time, changes in dietary habits are generally characterized by substitution effects, where high consumption of one component is associated with lower intake of other components [63]. This makes inferences about individual components particularly challenging. Food patterns pre-empt potential dietary confounding by other aspects of the diet, increase the ability to assess stronger effects due to the cumulative effects of many features of the diet, and allow assessment of the interaction among synergistic components. Even though observed associations with dietary patterns could be due to a single component rather than the overall dietary pattern, this can be tackled by performing food component analyses assessing the effect of single components for the overall association, as performed in the present study.

Strengths of this study include the prospective population-based design, which allowed for repeated eye examinations and thus prospectively ascertaining iOAG cases (according to a well-established OAG definition [26]), and IOP measurements. Moreover, we used validated FFQs to collect dietary data. Using dietary information from baseline assessments, we limited selection bias, since all included participants were free of OAG at this visit. Moreover, the FFQs were administered to cases and controls under similar conditions. Furthermore, we assessed the association between the MIND diet and iOAG over cumulative follow-up periods to provide insight into possible reversed causality. The persistence of the association over time implies that reverse causality is unlikely. Data on several possible confounders were available, although residual confounding cannot completely be ruled out. Given that our cases and controls were matched on age and sex, it is very unlikely that our findings were affected by the association of age and sex with dietary intake.

Limitations were also present. Using the prospective population-based design and including only participants with incident OAG, we limited the number of cases. Thus, there was insufficient statistical power for more detailed sub-group analyses (e.g., smokers vs. non-smokers). Using the FFQ, we relied on the participants’ memory for collecting information for as far back as 1 year. Also, participants are likely to under- or over-report certain foods. Since dietary data are collected before onset of disease, this would most likely lead to non-differential misclassification: participants that develop iOAG over time are expected to under- and over-report food items in a similar manner as participants that remain free of iOAG. Nevertheless, since 50% of all glaucoma is familial [64], it is possible that iOAG cases already adjusted their dietary patterns to anticipate their “expected” increased OAG risk. Nonetheless, this differential misclassification would result in a bias towards the null hypothesis, underestimating the true effect. Additionally, the data on dietary patterns that were collected at baseline do not per definition reflect long term intake as participants may change dietary habits over time. As dietary recommendations are not used as intervention for iOAG it is unlikely that participants who developed iOAG over time were more susceptible to dietary pattern changes than participants who did not develop iOAG. As a result, we do not expect that such non-differential misclassification would affect the associations present in our study. Lastly, treatment of chronic illnesses like hypertension and diabetes mellitus could have led to dietary changes during follow-up. However, since none of these illnesses are clearly associated with OAG [65, 66], this would probably result in non-differential misclassification, with limited effects on the associations found.

## Conclusion

In conclusion, we found an association between greater adherence to the MIND diet and lower risk of iOAG. The MIND diet was not associated with IOP, suggesting that its potential protective effect on iOAG is more likely via preserving retinal ganglion cells than through lowering IOP. Neither the Mediterranean diet nor the Dutch dietary guidelines were significantly associated with iOAG. Although the MIND diet appears to be promising for maintaining healthy eyes, these findings require confirmation.

## Supplementary Information

Below is the link to the electronic supplementary material.

Supplementary file 1 (DOCX 173 KB)
