# Supplementary material for: MIND diet lowers risk of open-angle glaucoma: the Rotterdam Study
Source: Eur J Nutr. 2022 Sep 20;62(1):477–87. doi: 10.1007/s00394-022-03003-w (PMC9899739; doi:10.1007/s00394-022-03003-w)
Supplement: Supplementary file 1 — (DOCX 173 KB) [file 394_2022_3003_MOESM1_ESM.docx]

**MIND diet lowers risk of open-angle glaucoma: the Rotterdam Study**

Joëlle E. Vergroesen (0000-0002-6093-4302), MSc^1,2^, Tosca O.E. de Crom (0000-0001-6869-043X), MSc^2^, Cornelia M. van Duijn (0000-0002-2374-9204), PhD^3^, Trudy Voortman (0000-0003-2830-6813), PhD^2,4^, Caroline C.W. Klaver (0000-0002-2355-5258), MD, PhD^1,2,5,6^, Wishal D. Ramdas (0000-0002-1780-5281), MD, PhD^1^

1) Department of Ophthalmology, Erasmus MC University Medical Center, PO Box 2040, 3000 CA Rotterdam, the Netherlands

2) Department of Epidemiology, Erasmus MC University Medical Center, PO Box 2040, 3000 CA Rotterdam, the Netherlands

3) Nuffield Department of Population Health, University of Oxford, OX3 7LF Oxford, United Kingdom

4) Division of Human Nutrition and Health, Wageningen University & Research, PO Box 17, 6700 AA Wageningen, the Netherlands

5) Department of Ophthalmology, Radboud University Medical Center, PO Box 9101, 6500 HB Nijmegen, the Netherlands

6) Institute of Molecular and Clinical Ophthalmology, University of Basel, Basel, Switzerland

**Corresponding author:**

Wishal D. Ramdas, MD, PhD: w.ramdas@erasmusmc.nl

**Journal:**

European Journal of Nutrition


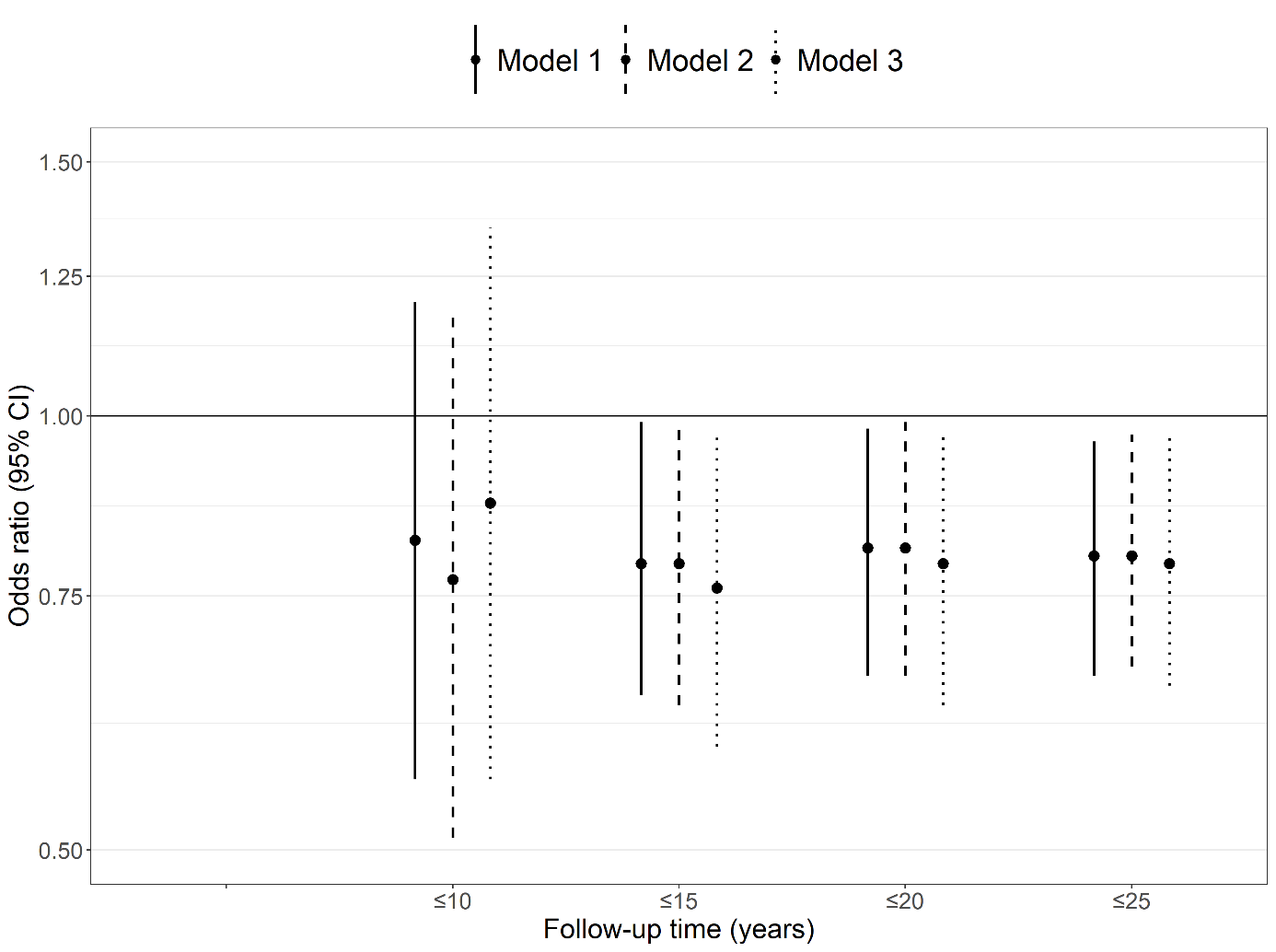


**Supplementary Figure 1.** Multivariable-adjusted odds ratios with corresponding 95% confidence intervals (CIs) for incident open-angle glaucoma per 10-percent increase in MIND diet adherence, shown per cumulative follow-up interval. Model 1: adjusted for body mass index, energy intake, physical activity, and follow-up time. Model 2: model 1 additionally adjusted for education level and smoking status. Model 3: model 1 additionally adjusted for intraocular pressure. Abbreviations: MIND = Mediterranean-DASH Intervention for Neurodegenerative Delay.

**Supplementary Table 1. Baseline characteristics of participants by adherence to the Mediterranean diet (per quartiles)**

|  | Q1 (N=239) | Q2 (N=359) | Q3 (N=109) | Q4 (N=313) | P ANOVA |
| --- | --- | --- | --- | --- | --- |
| iOAG, N (%) | 39 (16.3) | 60 (16.7) | 20 (18.3) | 51 (16.3) | 0.97 |
| Age (years) | 65.0 (7.3) | 66.1 (7.2) | 65.2 (6.3) | 63.8 (6.4) | <0.001* |
| Female sex, N (%) | 111 (46.4) | 199 (55.4) | 70 (64.2) | 172 (55.0) | 0.02* |
| Education, N (%)  Primary education  Lower education  Intermediate education  Higher education | 28 (11.7)  103 (43.1)  69 (28.9)  35 (14.6) | 56 (15.6)  164 (45.6)  92 (25.6)  44 (12.3) | 7 (6.4)  47 (43.1)  36 (33.0)  18 (16.5) | 35 (11.2)  128 (40.9)  86 (27.5)  64 (20.4) | 0.005* |
| Smoking status, N (%)  Non-smoker  Former smoker  Current smoker | 56 (23.4)  106 (44.4)  76 (31.8) | 120 (33.4)  166 (46.2)  71 (19.8) | 32 (29.4)  52 (47.7)  25 (22.9) | 106 (33.9)  160 (51.1)  47 (15.0) | <0.001* |
| BMI (kg/m^2^) | 27.2 (4.0) | 26.9 (4.1) | 26.6 (3.6) | 26.4 (3.9) | 0.10 |
| Energy intake (kcal/day) | 2038.7 (581.8) | 2033.9 (550.9) | 1942.5 (489.7) | 2265.3 (563.7) | <0.001* |
| Physical activity (MET hours/week) | 0.0 (1.0) | 0.0 (0.9) | 0.2 (1.0) | 0.1 (0.9) | 0.15 |
| IOP (mmHg) | 14.7 (3.3) | 14.7 (3.2) | 14.8 (3.6) | 14.3 (3.1) | 0.36 |
| Follow-up time (years) | 10.2 (5.0) | 10.2 (4.8) | 10.4 (5.1) | 9.2 (5.1) | 0.02* |
| Mediterranean diet adherence (%)^a,b^ | 48.9 (4.0) | 57.4 (2.0) | 61.8 (0.0) | 67.9 (4.6) | <0.001* |

Data are presented as mean (standard deviation), unless stated otherwise. ^a^ Percentage calculated from theoretical score range: 0-55; ^b^ Range: Q1: 29.1-52.7%, Q2: 52.7-60.0%, Q3: 60.0-61.8%, Q4: 61.8-90.9%; * P<0.05. Abbreviations: Q = quartile; ANOVA = analysis of variance; iOAG = incident open-angle glaucoma; N = number; SD = standard deviation; BMI = body mass index; MET = metabolic equivalent of task; IOP = intraocular pressure.

**Supplementary Table 2. Baseline characteristics of participants by adherence to the Dutch dietary guidelines (per quartiles)**

|  | Q1 (N=132) | Q2 (N=325) | Q3 (N=203) | Q4 (N=360) | P ANOVA |
| --- | --- | --- | --- | --- | --- |
| iOAG, N (%) | 23 (17.4) | 46 (14.2) | 33 (16.3) | 68 (18.9) | 0.42 |
| Age (years) | 64.6 (7.2) | 64.5 (7.0) | 64.9 (6.8) | 65.9 (7.0) | 0.06 |
| Female sex, N (%) | 54 (40.9) | 142 (43.7) | 117 (57.6) | 239 (66.4) | <0.001* |
| Education, N (%)  Primary education  Lower education  Intermediate education  Higher education | 9 (6.8)  54 (40.9)  38 (28.8)  30 (22.7) | 41 (12.6)  140 (43.1)  95 (29.2)  45 (13.8) | 31 (15.3)  89 (43.8)  54 (26.6)  26 (12.8) | 45 (12.5)  159 (44.2)  96 (26.7)  60 (16.7) | 0.02* |
| Smoking status, N (%)  Non-smoker  Former smoker  Current smoker | 24 (18.2)  62 (47.0)  46 (34.8) | 79 (24.3)  160 (49.2)  83 (25.5) | 68 (33.5)  96 (47.3)  39 (19.2) | 143 (39.7)  166 (46.1)  51 (14.2) | <0.001* |
| BMI (kg/m^2^) | 27.6 (4.3) | 26.9 (3.7) | 26.7 (4.0) | 26.5 (4.1) | 0.03* |
| Energy intake (kcal/day) | 2078.6 (539.5) | 2140.1 (597.7) | 2060.8 (580.5) | 2083.1 (540.9) | 0.38 |
| Physical activity (MET hours/week) | 0.0 (1.0) | 0.0 (0.9) | 0.1 (0.9) | 0.1 (1.0) | 0.27 |
| IOP (mmHg) | 14.7 (3.4) | 14.6 (3.1) | 14.5 (3.1) | 14.6 (3.3) | 0.94 |
| Follow-up time (years) | 10.0 (4.9) | 10.0 (5.0) | 10.0 (5.1) | 9.8 (4.9) | 0.92 |
| Dutch dietary guidelines adherence^a,b^ | 25.4 (4.5) | 40.0 (3.5) | 50.0 (0.0) | 62.6 (6.6) | <0.001* |

Data are presented as mean (standard deviation), unless stated otherwise. ^a^ Percentage calculated from theoretical score range: 0-14; ^b^ Range: Q1: 7.1-28.6%, Q2: 28.6-42.9%, Q3: 42.9-50.0%, Q4; 50.0-92.9%; * P<0.05. Abbreviations: Q = quartile; ANOVA = analysis of variance; iOAG = incident open-angle glaucoma; N = number; SD = standard deviation; BMI = body mass index; MET = metabolic equivalent of task; IOP = intraocular pressure.

**Supplementary Table 3. Multivariable-adjusted odds ratios with corresponding 95% confidence intervals for incident open-angle glaucoma per 10-percent increase in MIND diet adherence, and food component analyses**

|  | Model 1 | | Model 2 | | Model 3 | |
| --- | --- | --- | --- | --- | --- | --- |
|  | Odds ratio | P-value | Odds ratio | P-value | Odds ratio | P-value |
| Total MIND diet adherence | 0.80 (0.66; 0.96) | 0.02 | 0.80 (0.67; 0.97) | 0.02 | 0.79 (0.65; 0.97) | 0.02 |
| Minus wine | 0.80 (0.67; 0.97) | 0.02 | 0.80 (0.66; 0.98) | 0.03 | 0.81 (0.66; 0.99) | 0.04 |
| Minus whole grains | 0.77 (0.63; 0.93) | 0.007 | 0.77 (0.63; 0.93) | 0.008 | 0.75 (0.61; 0.92) | 0.007 |
| Minus red meats | 0.75 (0.61; 0.92) | 0.005 | 0.76 (0.62; 0.93) | 0.007 | 0.75 (0.61; 0.94) | 0.01 |
| Minus poultry | 0.82 (0.68; 1.00) | 0.05 | 0.83 (0.68; 1.02) | 0.07 | 0.82 (0.66; 1.01) | 0.07 |
| Minus pastries and sweets | 0.75 (0.62; 0.92) | 0.005 | 0.75 (0.61; 0.92) | 0.006 | 0.74 (0.59; 0.92) | 0.006 |
| Minus other vegetables | 0.78 (0.64; 0.94) | 0.008 | 0.78 (0.64; 0.94) | 0.01 | 0.78 (0.64; 0.96) | 0.02 |
| Minus olive oil | 0.82 (0.68; 0.99) | 0.04 | 0.82 (0.67; 0.99) | 0.04 | 0.82 (0.67; 1.01) | 0.06 |
| Minus nuts | 0.77 (0.64; 0.94) | 0.009 | 0.77 (0.63; 0.94) | 0.01 | 0.77 (0.62; 0.95) | 0.02 |
| Minus green leafy vegetables* | 0.86 (0.71; 1.05) | 0.14 | 0.86 (0.70; 1.05) | 0.15 | 0.87 (0.70; 1.08) | 0.20 |
| Minus fried/fast food | 0.78 (0.64; 0.94) | 0.009 | 0.78 (0.64; 0.94) | 0.01 | 0.78 (0.63; 0.96) | 0.02 |
| Minus fish* | 0.84 (0.68; 1.05) | 0.12 | 0.84 (0.68; 1.05) | 0.12 | 0.83 (0.65; 1.04) | 0.11 |
| Minus cheese | 0.80 (0.66; 0.97) | 0.02 | 0.80 (0.66; 0.97) | 0.03 | 0.78 (0.64; 0.96) | 0.02 |
| Minus butter and stick  margarine | 0.81 (0.67; 0.99) | 0.04 | 0.82 (0.67; 1.00) | 0.05 | 0.81 (0.65; 0.99) | 0.04 |
| Minus berries* | 0.89 (0.73; 1.08) | 0.24 | 0.89 (0.73; 1.08) | 0.23 | 0.87 (0.70; 1.07) | 0.19 |
| Minus beans | 0.82 (0.68; 1.00) | 0.05 | 0.83 (0.68; 1.01) | 0.06 | 0.80 (0.65; 1.00) | 0.05 |

Model 1: adjusted for body mass index, energy intake, physical activity, follow-up time and MIND diet adherence of components of interest. Model 2: model 1 additionally adjusted for education level and smoking status. Model 3: model 1 additionally adjusted for intraocular pressure. * The association between adherence to the MIND and incident open-angle glaucoma substantially changed after excluding this particular component adherence. Abbreviations: MIND = Mediterranean-DASH Intervention for Neurodegenerative Delay.

**Supplementary Table 4. Multivariable-adjusted beta’s with corresponding 95% confidence intervals for intraocular pressure per 1-unit increase in food component**

|  | Model 1 | | Model 2 | |
| --- | --- | --- | --- | --- |
|  | Beta | P-value | Beta | P-value |
| Green leafy vegetables (250 g/week) | 0.05 (-0.22; 0.31) | 0.73 | 0.05 (-0.21; 0.32) | 0.69 |
| Berries (50 g/week) | -0.11 (-0.21; -0.01) | 0.04* | -0.11 (-0.21; 0.00) | 0.05 |
| Fish (250 g/month) | -0.01 (-0.10; 0.09) | 0.88 | -0.02 (-0.11; 0.08) | 0.75 |

Model 1: adjusted for body mass index, energy intake, physical activity, and follow-up time. Model 2: model 1 additionally adjusted for education level and smoking status. * P<0.05
